# Immobilization by 21-days of bed rest causes changes in biomarkers of cartilage homeostasis in healthy individuals

**DOI:** 10.1016/j.ocarto.2025.100597

**Published:** 2025-02-28

**Authors:** Maren Dreiner, Elie-Tino Godonou, Annegret Mündermann, Koray Tascilar, Georg Schett, Frank Zaucke, Anna-Maria Liphardt, Anja Niehoff

**Affiliations:** a Institute of Biomechanics and Orthopaedics, German Sport University Cologne, Köln, Germany; b Department of Internal Medicine – Rheumatology & Immunology, Universitätsklinikum Erlangen & Friedrich-Alexander-Universität Erlangen-Nürnberg, Erlangen, Germany; c Deutsches Zentrum Immuntherapie (DZI), Universitätsklinikum Erlangen & Friedrich-Alexander-Universität Erlangen-Nürnberg, Erlangen, Germany; d Department of Teaching, Research and Development, Schulthess Clinic, Zurich, Switzerland; e Department of Biomedical Engineering, University of Basel, Basel, Switzerland; f Department of Clinical Research, University of Basel, Basel, Switzerland; g Dr. Rolf M. Schwiete Research Unit for Osteoarthritis, Department of Trauma Surgery and Orthopaedics, University Hospital, Goethe University Frankfurt, Frankfurt, Germany; h Cologne Center for Musculoskeletal Biomechanics (CCMB), Medical Faculty, University of Cologne, Köln, Germany

**Keywords:** Non-collagenous biomarkers, Cartilage homeostasis, Cartilage biomarkers, Immobilization, Bed rest, MNX-Study

## Abstract

**Objective:**

To investigate the effects of 21 days bed rest immobilization (with and without exercise and nutrition intervention) on serum concentrations of cartilage homeostasis biomarkers in healthy individuals.

**Design:**

Twelve male volunteers (age 34.2 ​± ​8.3 years; BMI 22.4 ​± ​1.7 ​kg/m^2^) participated in 6 days of baseline data collection (BDC), 21 days of 6° head-down-tilt (HDT) bed rest (CON) ​+ ​interventions HDT ​+ ​resistive vibration exercise (RVE; 2 times/week; 25 ​min) and HDT ​+ ​RVE ​+ ​nutrition (NeX; 0.6 ​g/kg body weight/day whey protein and 90 ​mmol KHCO_3_/day bicarbonate supplementation), and 6 days of recovery (R) in a cross-over designed study. The starting HDT condition was randomized (CON-RVE-NeX, RVE-NeX-CON, NeX-CON-RVE). Blood samples were collected before, during and after HDT. Serum concentrations of COMP, MMP-3, MMP-9, YKL-40 and resistin were analyzed.

**Results:**

The main effect of time was significant for all biomarkers tested (p ​< ​0.001). While COMP (−36 ​% at HDT5, p ​< ​0.001) and MMP-3 (−36 ​% at HDT21, p ​< ​0.001) decreased during HDT bed rest, MMP-9 (+18 ​% at HDT5, p ​< ​0.001) and resistin (+13 ​% at HDT21, p ​< ​0.001) increased during HDT bed rest. Interestingly, during recovery, YKL-40 levels increased (+13 ​% at R1, p ​= ​0.022), while MMP-9 levels decreased (−19 ​% at R6, p ​= ​0.035). We identified correlations between COMP and MMP-3 (r_rm_ ​= ​0.58, p ​< ​0.001) as well as between MMP-9 and resistin (r_rm_ ​= ​0.58, p ​< ​0.001).

**Conclusions:**

Immobilization affects serum concentrations of cartilage homeostasis biomarkers suggesting changes in cartilage metabolism that do not completely recover during re-ambulation. Both interventions had only minimal effects.

## 1 Introduction

Mechanical loading is an important contributor in the initiation of abnormal joint tissue metabolism [1] and mechanical overloading or disuse can alter cartilage homeostasis [2]. Biochemical markers (biomarkers) in blood, urine or synovial fluid reflect homeostasis of articular tissues [3]. They can thus aid in the diagnosis and prognosis of cartilage-related diseases and enable monitoring of disease progression and treatment efficacy [4]. We have previously reported a shift towards cartilage degradation in response to immobilization based on the kinetics of type II collagen related biomarkers during 21 days of bed rest in healthy men [5]. Here, we extended our investigations to a set of non-collagenous biomarkers known to reflect anabolic and catabolic processes in the cartilage extracellular matrix (ECM).

Cartilage oligomeric matrix protein (COMP, or thrombospondin (TSP)-5) is an integral component of the cartilage ECM and crucial for its assembly and stability [6]. Matrix metalloproteinases (MMPs) are a large family of extracellular zinc-dependent endopeptidases involved in both the synthesis and breakdown of ECM components [7]. MMP-3 (stromelysin-1) is involved in cartilage degradation [8] and can activate other MMPs such as MMP-9 [9]. YKL-40 (also called chitinase-3-like protein (CHI3L1)) is associated with inflammation, tissue remodeling and angiogenesis of articular cartilage [10]. Resistin is a cysteine-rich polypeptide hormone protein that has been suggested to mediate inflammation and to play a role not only in obesity, insulin resistance and diabetes [11], but also in rheumatoid arthritis [12] and OA [13]. Interestingly, some of these biomarkers (including COMP and MMP-3) are highly sensitive to physical activity [14] and inactivity [15].

Cartilage atrophy (thinning of human articular cartilage), has been described in response to joint unloading [16,17]. In addition, periods of limb immobilization in a mouse model resulted in thinner cartilage with lower aggrecan content [18] and decreased cartilage stiffness [19]. Decreasing levels of physical activity may also contribute to the rising prevalence of articular cartilage degeneration in humans, resulting in weaker, more unstable and less resistant joints [20]. Generally, immobilization (e.g. due to injury or illness) and reduced physical activity levels are risk factors for OA [21].

Mechanical loading supports the maintenance of physiological articular cartilage metabolism and joint integrity by enabling the influx of nutrients, the efflux of breakdown products and their exchange between surrounding joint structures [22]. Physical exercise may be beneficial for articular cartilage, induce a biochemical response [23] and may have therapeutic effects in patients with OA [24], but the optimal exercise protocol remains unclear. Whole body vibration training [25] has been discussed as an exercise mode with the advantages of short training sessions and the simultaneous stimulation of all musculoskeletal tissues.

Diet is another important factor in musculoskeletal homeostasis and the range of dietary sources (natural compounds and nutraceuticals) that may affects joint health is broad [26]. Protein supplementation after resistance exercise can increase muscle mass and strength compared to placebo interventions [27]. However, while beneficial for muscle, protein may increase bone resorption [28]. Alkaline mineral salts, such as potassium bicarbonate, counteract acidosis and improve bone mineral density [29].

The contribution of lifestyle factors such as physical activity and diet to cartilage atrophy is difficult to investigate in larger populations or cohorts. 6° head-down-tilt (HDT) bed rest is a highly standardized and established model [30,31] (especially to simulate the effects of microgravity on Earth) that allows characterization of the effect of immobilization on cartilage metabolism in healthy individuals in a highly standardized setting.

The purpose of this study was (I) to investigate the effect of 21 days of HDT bed rest on serum concentrations of cartilage homeostasis biomarkers (COMP, MMP-3, MMP-9, YKL-40 and resistin), (II) to analyze the interaction between these biomarkers in response to HDT bed rest, and (III) to evaluate the potential of resistive vibration exercise (RVE) and RVE in combination with a nutritional countermeasure (whey and bicarbonate supplementation) (NeX) to modulate the bed rest response. We hypothesized that 21 days of HDT bed rest will (I) result in changes in serum concentrations of COMP, MMP-3, MMP-9, YKL-40 and resistin, (II) there will be an interaction between these biomarkers in response to HDT bed rest, and (III) the countermeasures applied will affect the biomarker response.

## 2 Material and methods

The “Medium duration nutrition and vibration exercise” (MNX) study was conducted by the European Space Agency (ESA) in 2012 and 2013 ​at the Institute for Space Medicine and Physiology (MEDES Clinique d'Investigation, Toulouse, France, ID-RCB: 2012-A00337-36). The study was approved by the French Health Authorities (Ethics Committee: CPP Sud-Ouest Outre-Mer I) and conducted according to the guidelines of the Declaration of Helsinki (1989). Details of the study design have been published previously [5,[32], [33], [34], [35]] and only basic information for this analysis is summarized below. The data and analyses presented here complement our previous analysis of changes in type II collagen biomarker concentrations in the MNX study [5].

### 2.1 Participants

Twelve healthy men were enrolled in the study (Table 1) after giving written informed consent. A comprehensive medical and psychological screening process was performed at MEDES prior to participation, including medical history, physical and psychological examinations, laboratory parameters, and microbiological screening [5].

**Table 1:** Baseline participant characteristics campaign 1 (mean ​± ​standard deviation). BMI: body mass index.

Baseline characteristics	Mean ​± ​SD
Age [years]	34.2 ​± ​8.3
Height [cm]	176 ​± ​6
Body mass [kg]	69.8 ​± ​8.0
BMI [kg/m^2^]	22.4 ​± ​1.7

### 2.2 Study design

The study was conducted in a cross-over design (all participants completed all interventions, Table S1) with three identically designed campaigns consisting of the same study periods (Fig. 1): 6 days of baseline data collection (BDC-6 to BDC-1), 21 days of HDT bed rest with intervention (HDT1 to HDT21), and 6 days of recovery (R1 to R6). The “wash-out” period between the study campaigns was 4 months, resulting in a total study duration of 1 year.

**Fig. 1:**
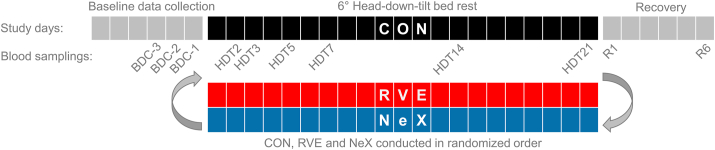
Cross-over study design consisting of three campaigns of 30 days each. Each campaign included 6 days baseline data collection (BDC), 21 days of head-down-tilt (HDT) bed rest, and 6 days of recovery (R). During the 21 days of HDT bed rest, the interventions were applied in a randomized order: control intervention (CON), resistive vibration exercise (RVE) intervention, and nutrition ​+ ​RVE (NeX) intervention .

During the intervention period, participants spent 24 ​h/day in the HDT bed rest position (supine, lateral, or prone) in double rooms. HDT stretchers were used for transportation to the experiments, and video monitoring was used to ensure compliance with HDT bed rest. A standardized diet tailored to the individual basal resting metabolic rate (determined by a subject-specific normo-caloric diet during BDC) was maintained in all study campaigns (see previous publications for details [5,32]).

### 2.3 Interventions

During the HDT bed rest periods, three different interventions were applied [32] as predefined by the European Space Agency.

• Control (CON): 21 days of HDT bed rest without additional interventions.
• Resistive vibration exercise (RVE): 25-min leg muscle resistive vibration exercise twice per week (HDT2, HDT5, HDT9, HDT12, HDT16, and HDT21) in the HDT bed rest position on the Galileo Space exercise device [32] (Novotec Medical GmbH). During the resistive vibration exercise, participants placed their feet on a side-alternating vibration plate and moved the backrest against a predefined force (approximately two times body weight) provided by a pneumatic system. The vibration load was set to an amplitude of 8 ​mm and a frequency of 25 ​Hz (reduced to 16 ​Hz for toe raises).
• RVE ​+ ​nutritional supplementation (NeX): RVE protocol combined with isocaloric nutritional supplementation (whey protein (0.6 ​g/kg body weight/day; total protein intake of 1.8 ​g/kg body weight/day) and potassium bicarbonate (90 ​mmol KHCO_3_/day) (for details see Ref. [32])).

Participants were required to complete all campaigns and were randomized to the starting condition, resulting in the following orders of interventions: CON-RVE-NeX, RVE-NeX-CON, NeX-CON-RVE.

### 2.4 Blood sampling and biomarker analysis

Venous blood samples were collected from the antecubital vein using a 7.5 ​mL serum Monovette® (Sarstedt, Germany) after overnight fasting and rest (approximately 8 ​h) on the following study days: BDC-3, BDC-2, BDC-1; HDT2, HDT3, HDT5, HDT7, HDT14, HDT21; R1 and R6 (Fig. 1). Blood sampling conditions were identical in all study periods and all study campaigns. After 30 ​min of clotting, the blood was centrifuged at 2000×*g* for 10 ​min at room temperature, serum was aliquoted in freezing tubes and frozen at −80 ​°C until analysis. Serum concentrations of all biomarkers were analyzed using commercially available enzyme-linked immunosorbent assays (ELISAs) (Table S2). All serum samples from the same participant were analyzed in duplicates on the same ELISA plate, using ELISA kits from the same lot for each respective biomarker. The intra- and inter-assay coefficients of variability for each biomarker are reported in Table S2.

### 2.5 Statistical analysis

Biomarker results are presented as mean (95 ​% confidence interval, CI), and we performed all statistical analyses using R (version 4.1.2). To identify significant changes in serum biomarker concentrations over time, we used linear mixed-effect models (LMM) that included time points and interventions as covariates. An interaction term including time points and interventions was included in all models and retained if statistically significant. To calculate differences in the response to the study interventions, we used estimated marginal means derived from the mixed models. We used the Restricted Maximum Likelihood (REML) approach for model estimation, with subjects serving as a random effect factor. Given the clustered structure of the inherent in linear mixed models, we employed robust estimation of standard errors using the Robust Covariance Matrix Estimation for Mixed Models. Model comparisons were based on Akaike's Information Criterion (AIC) [36]. To assess the goodness of fit of the LMM, we computed R^2^ values using Nagelkerke's generalized R [2,37] and reported the Intraclass Correlation (ICC) of the models [38]. The predetermined significance level for all statistical tests was set a priori at 0.05.

In this study, each subject had a predetermined number of measurements taken at standardized time points. However, four participants left the study before data collection was completed, resulting in missing observations. Consequently, we had 12 data sets, but only eight subjects had complete data for all time points. LMMs using the Restricted Maximum Likelihood method [39] were used because they can handle missing data and utilize most, if not all, of the experimental data without excluding participants.

We investigated the correlation between the biomarkers by analyzing the biomarker data at the intra-individual concentration (longitudinal change) using repeated measures correlation matrix [40]; p-values were adjusted for multiple comparisons using the False Discovery Rate (FDR) method. An LMM was then used to examine the relationship between the time series of COMP concentration and the time series of MMP-3, MMP-9, YKL-40 and resistin concentration; this model included MMP-3, MMP-9, YKL-40, and resistin as predictors while adjusting for “intervention” covariate.

## 3 Results

**Fig. 2:**
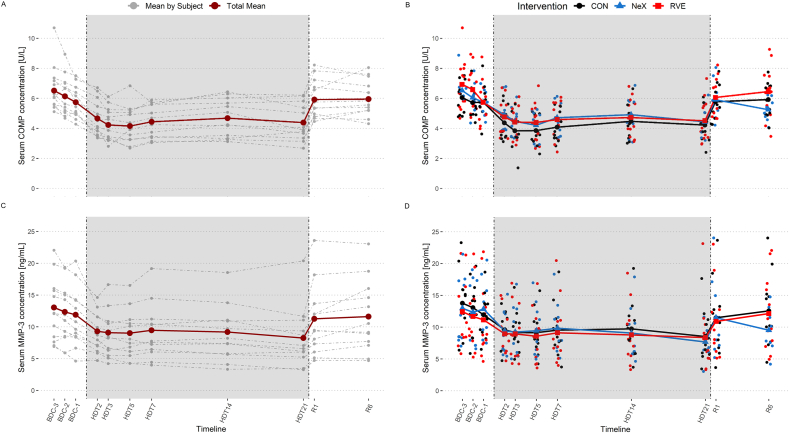
Absolute serum concentrations of COMP (A, B) and MMP-3 (C, D) over time. Left panel (A and C): mean of all interventions for each subject (gray-dashed lines) and overall mean for all subjects and interventions (red lines). Right panel (B and D): mean by intervention over time (colored solid lines: intervention means; colored dots: individual values of subjects). BDC: baseline data collection; CON: control intervention; HDT: head-down-tilt bed rest; RVE: resistive vibration exercise; NeX: nutrition and RVE; R: recovery.

In each of the campaigns, COMP concentrations were highest at BDC-3 compared to all other time points (Fig. 2A and B), and values already decreased from BDC-3 to BDC-1. During the first week of HDT bed rest, COMP concentrations decreased to the lowest value at HDT3 and remained low until HDT21. With the start of the recovery (R1), COMP concentrations increased again (Fig. S1, concentrations normalized to baseline). Comparing the interventions, COMP concentrations were lower during HDT bed rest for the control intervention (CON) intervention. LMM analysis showed a main effect of time (p ​< ​0.001), intervention (p ​< ​0.001), and their interaction (time ∗ intervention, p ​= ​0.025) (Table 2). COMP concentrations were reduced compared to baseline (BDC-3) at all time points during HDT bed rest (HDT2, HDT3, HDT5, HDT7, HDT14, HDT21, p ​< ​0.001), with a reduction of −2.21 ​U/L at HDT3 (p ​< ​0.001) (Table 3). While there were no statistically significant differences in COMP concentrations between interventions during the HDT bed rest, values were lower −1.14 ​U/L (p ​= ​0.002) at time point R6 for the NeX intervention compared to CON.

**Table 2:** Summaries of LMMs for biomarkers over time and by intervention. COMP: cartilage oligomeric matrix protein, Df: degrees of freedom, LMM: linear mixed model, MMP: matrix metalloproteinase and Χ^2^: chi-square.

	COMP			MMP-3			MMP-9			YKL-40			Resistin		
	Χ^2^	Df	p	Χ^2^	Df	p	Χ^2^	Df	p	Χ^2^	Df	p	Χ^2^	Df	P
Time	683.66	10	<**0.001**	312.55	10	<**0.001**	99.03	10	<**0.001**	51.81	10	<**0.001**	60.37	10	<**0.001**
Intervention	15.16	2	<**0.001**	3.66	2	0.160	3.45	2	0.178	1.75	2	0.417	1.12	2	0.572
Time:Intervention	34.22	20	**0.025**	16.21	20	0.703	14.05	20	0.828	13.02	20	0.877	24.05	20	0.240

**Table 3:** Fixed effect of LMM to detect significant changes in serum biomarkers (COMP, MMP-3, MMP-9, YKL-40 and resistin) separated by time point and intervention. BDC: baseline data collection, CI: 95 ​% confidence interval, COMP: cartilage oligomeric matrix protein, CON: control intervention, Conditional R^2^: considers both fixed and random effects, HDT: head-down-tilt bed rest, ICC: intraclass correlation coefficient, LMM: linear mixed model, marginal R^2^: variance of the fixed effects, MMP: matrix metalloproteinase, N: number of participants, NeX: nutrition and RVE, RVE: resistive vibration exercise, R: recovery, Ref.: Reference level, σ^2^: variance and τ_00_: between-participant variance.

	COMP		MMP-3		MMP-9		YKL-40		Resistin	
Predictors	Estimates (CI)	p	Estimates (CI)	p	Estimates (CI)	p	Estimates (CI)	p	Estimates (CI)	p
Intercept	6.22 (5.54 to 6.90)	<**0.001**	13.28 (10.62 to 15.94)	<**0.001**	378.8 (299.9 to 457.7)	<**0.001**	39.69 (29.39 to 49.99)	<**0.001**	7.84 (5.57 to 10.12)	<**0.001**
Time point										
BDC-3	Ref.		Ref.		Ref.		Ref.		Ref.	
BDC-2	−0.33 (−0.74 to 0.07)	0.108	−0.70 (−2.08 to 0.69)	0.083	24.6 (−15.4 to 64.6)	0.227	−1.64 (−4.08 to 0.79)	0.185	−0.10 (−0.48 to 0.27)	0.583
BDC-1	−0.30 (−0.75 to 0.15)	0.186	−1.82 (−3.20 to 0.44)	**0.022**	86.9 (−14.5 to 188.2)	0.093	−2.62 (−6.39 to 1.16)	0.173	0.50 (−1.02 to 2.02)	0.521
HDT2	−1.68 (−2.09 to 1.27)	<**0.001**	−4.17 (−5.56 to 2.79)	<**0.001**	65.0 (3.4 to 126.5)	**0.039**	1.39 (−2.88 to 5.65)	0.523	0.17 (−0.32 to 0.65)	0.504
HDT3	−2.21 (−2.86 to 1.56)	<**0.001**	−4.62 (−6.01 to 3.24)	<**0.001**	62.5 (21.0–103.9)	**0.003**	−0.87 (−3.86 to 2.11)	0.565	−0.02 (−0.56 to 0.51)	0.928
HDT5	−2.20 (−2.66 to 1.73)	<**0.001**	−4.64 (−6.02 to 3.25)	<**0.001**	91.9 (62.6 to 121.1)	<**0.001**	−3.29 (−7.07 to 0.49)	0.088	0.32 (−0.26 to 0.90)	0.275
HDT7	−1.97 (−2.34 to 1.60)	<**0.001**	−4.24 (−5.62 to 2.86)	<**0.001**	45.3 (2.4 to 88.1)	**0.038**	−0.09 (−4.87 to 4.68)	0.969	0.25 (−0.63 to 1.13)	0.578
HDT14	−1.58 (−2.00 to 1.16)	<**0.001**	−4.04 (−5.42 to 2.66)	<**0.001**	40.9 (1.3 to 80.6)	**0.043**	−1.14 (−4.96 to 2.68)	0.556	0.96 (0.34 to 1.58)	**0.002**
HDT21	−1.83 (−2.22 to 1.43)	<**0.001**	−5.24 (−6.62 to 3.86)	<**0.001**	28.3 (−27.5 to 84.1)	0.320	3.39 (−2.08 to 8.87)	0.224	1.28 (0.74 to 1.82)	<**0.001**
R1	−0.27 (−0.77 to 0.23)	0.288	−2.30 (−3.68 to 0.92)	**0.020**	24.2 (−21.5 to 69.8)	0.298	8.05 (1.15 to 14.95)	**0.022**	0.14 (−0.52 to 0.81)	0.674
R6	−0.13 (−0.52 to 0.26)	0.518	−1.21 (−2.59 to 0.17)	0.148	−56.6 (−109.2 to 4.0)	**0.035**	0.22 (−5.74 to 6.18)	0.942	−0.59 (−1.28 to 0.11)	0.097
Intervention										
CON	Ref.		Ref.		Ref.		Ref.		Ref.	
NeX	0.30 (−0.34 to 0.93)	0.359	−1.03 (−2.49 to 0.43)	0.220	44.2 (−35.5 to 123.9)	0.276	2.52 (−0.17 to 5.22)	0.066	0.91 (−0.25 to 2.08)	0.125
RVE	0.71 (−0.10 to 1.53)	0.087	−0.85 (−2.20 to 0.51)	0.254	30.3 (−8.3 to 68.8)	0.123	1.23 (−4.05 to 6.51)	0.648	0.30 (−0.33 to 0.93)	0.352
σ^2^	0.35		2.71		5599.6		41.09		1.05	
τ_00_	1.31 _Subject_		18.96 _Subject_		23110.6 _Subject_		211.45 _Subject_		11.62 _Subject_	
ICC	0.79		0.87		0.80		0.84		0.92	
N	12 _Subject_		12 _Subject_		12 _Subject_		12 _Subject_		12 _Subject_	
Observations	341		350		349		348		349	
Marginal R^2^/Conditional R^2^	0.315/0.854		0.107/0.888		0.062/0.817		0.030/0.842		0.020/0.919	

The kinetics of MMP-3 were similar to those of COMP, with the highest MMP-3 concentration at BDC-3 for all campaigns compared to all other time points. MMP-3 started to decrease already during the baseline data collection (BDC) phase in all campaigns (Fig. 2C and D). While the decrease in MMP-3 concentration was most pronounced after 48 ​h of bed rest (BDC-1 to HDT2), the lowest values were measured on HDT21. With the start of recovery, MMP-3 concentrations started to increase again. LMM analysis revealed a main effect of time (p ​< ​0.001) but no effect of intervention (p ​= ​0.160) or interaction (time ∗ intervention, p ​= ​0.703) on MMP-3 concentrations. Already during the BDC phase, MMP-3 concentrations decreased with an average reduction of −1.82 ​ng/mL between BDC-3 and BDC-1 (p ​= ​0.022). Concentrations at all HDT bed rest time points (HDT2, HDT3, HDT5, HDT7, HDT14 and HDT21) were lower (p ​< ​0.001) compared to baseline (BDC-3). On the first day of recovery, MMP-3 concentrations increased, but were still lower (−2.30 ​ng/mL, p ​= ​0.020) compared to baseline (BDC-3). MMP-3 concentrations reached baseline concentrations on R6 (−1.21 ​ng/mL, p ​= ​0.148).

**Fig. 3:**
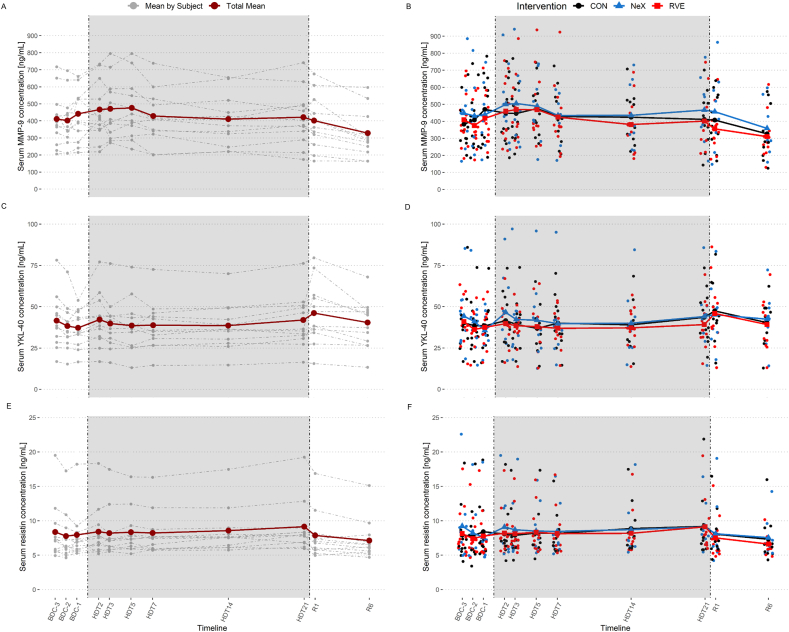
Absolute serum concentrations of MMP-9 (A, B), YKL-40 (C, D) and resistin (E, F) over time. Left panel (A, C and E): mean of all interventions for each subject (gray-dashed lines) and overall mean for all subjects and interventions (red lines). Right panel (B, D and F): mean by intervention over time (colored solid lines: intervention means; colored dots: individual values of subjects). BDC: baseline data collection; CON: control intervention; HDT: head-down-tilt bed rest; RVE: resistive vibration exercise; NeX: nutrition and RVE; R: recovery.

MMP-9 concentrations increased from BDC-2 to BDC-1 and, in contrast to COMP and MMP-3, continued to increase during HDT bed rest, peaking at HDT5 (Fig. 3A and B). After HDT5, MMP-9 concentrations decreased until HDT14 and showed a pronounced reduction below baseline values between R1 and R6. The LMM analysis showed a main effect of time (p ​< ​0.001), but no effect of intervention (p ​= ​0.178) or interaction (time point ∗ intervention, p ​= ​0.828). MMP-9 concentrations were higher compared to baseline at most HDT bed rest time points (HDT2, HDT3, HDT5, HDT7, HDT14) (p ​< ​0.05, Table 3), except for the time point HDT21 (p ​= ​0.320). Concentrations were reduced (−56.6 ​ng/mL, p ​= ​0.035) on the last time point R6 compared to baseline.

YKL-40 concentrations remained stable during BDC and HDT bed rest, except for an increase at HDT2, which appeared to be more pronounced for the NeX intervention (Fig. 3C and D). LMM analysis revealed a main effect of time (p ​< ​0.001) with YKL-40 concentrations being higher at R1 compared to BDC-3 (+8.05 ​ng/mL, p ​= ​0.022). No effect of the intervention (p ​= ​0.417) or the interaction (time ∗ intervention, p ​= ​0.877) was detected.

Resistin concentrations increased slightly at the start of HDT bed rest, became more pronounced towards the end of HDT bed rest, and returned to baseline concentrations during recovery. LMM analysis revealed a main effect of time (p ​< ​0.001) with increased resistin concentrations compared to baseline at HDT14 (+0.96 ​ng/mL, p ​= ​0.002) and HDT21 (+1.28 ​ng/mL, p ​< ​0.001) (Fig. 3E and F). No effect of the intervention (p ​= ​0.572) or the interaction (time ∗ intervention, p ​= ​0.240) was detected.

**Fig. 4:**
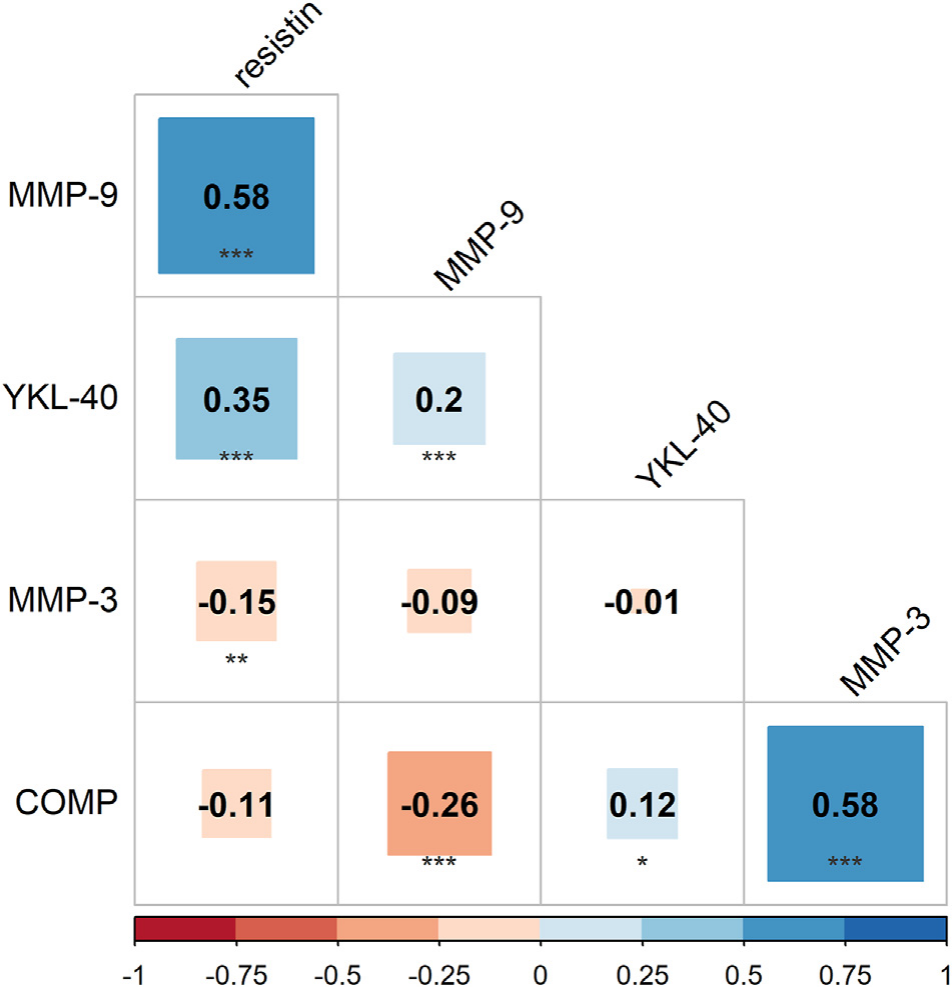
Plot of repeated measures correlation matrix between time series of different biomarkers with adjusted p-values. ∗p ​< ​0.05, ∗∗p ​< ​0.01 and ∗∗∗p ​< ​0.001.

We observed a positive correlation between the time series of COMP and MMP-3 concentrations (r_rm_ ​= ​0.58, 95 ​% CI [0.50, 0.65], p ​< ​0.001), i.e. as COMP concentrations decreased during HDT bed rest, MMP-3 also tended to decrease. Another positive correlation was found between the time series of MMP-9 and resistin concentrations (r_rm_ ​= ​0.58, 95 ​% CI [0.50, 0.65], p ​< ​0.001). Both biomarkers increased during HDT bed rest. Negative correlations were observed between the time series of COMP and MMP-9 concentrations (r_rm_ ​= ​−0.26, 95 ​% CI [−0.35, −0.15], p ​< ​0.001) and between the time series of MMP-3 and resistin concentrations (r_rm_ ​= ​−0.15, 95 ​% CI [−0.25, −0.04], p ​= ​0.01). Additional correlation results are shown in Fig. 4 and Table S3.

The results of the LMM analysis (Table S4) examining the association between the biomarkers were very similar to the repeated measures correlation results. Changes in COMP over time were associated with changes in MMP-3, MMP-9 and YKL-40 over time. For each 1-unit increase in MMP-3 and YKL-40 concentrations, the predicted value of COMP concentration increased significantly by 0.27 (p ​< ​0.001) and 0.03 (p ​< ​0.001), respectively, whereas it decreased slightly by −0.004 for each 1-unit increase in MMP-9 concentration, assuming all other predictor variables or covariates were held constant.

## 4 Discussion

In this study, we quantified the serum concentrations of five biomarkers reflecting cartilage homeostasis (COMP, MMP-3, MMP-9, YKL-40 and resistin) before, during and after 21-days of HDT bed rest in healthy men and evaluated the effects of exercise and nutrition countermeasures (RVE and NeX) during the HDT bed rest. The overall results indicate a metabolic response to HDT bed rest, evident by changes in all selected biomarkers, with varying magnitude and direction and at different time points. Concentrations of mechanosensitive biomarkers such as COMP and MMP-3 were reduced during HDT bed rest. In contrast, concentrations of MMP-9 and resistin were increased at various time points during HDT bed rest. COMP was the only biomarker that was affected by the countermeasures, albeit minimally, with a reduction of −1.14 ​U/L for the NeX intervention compared to control in the recovery phase (R6).

Previous studies have shown that serum COMP and MMP-3 concentrations are similarly affected by HDT bed rest [15,25]. For example, in a 14-day bed rest study [25], COMP concentrations decreased on average by 14.8 ​% during HDT bed rest (control intervention), and a whole body vibration training intervention did not counteract this effect. Our findings for COMP, MMP-3 and MMP-9 are also consistent with results from a previous 21-day HDT bed rest study [15]. Nutritional countermeasures (potassium bicarbonate supplementation; whey protein ​+ ​potassium bicarbonate) did not affect the outcomes [15]. The decrease in COMP and MMP-3 concentrations during bed rest is likely attributable to the mechanosensitivity of these biomarkers [14], transport rates of metabolites out of the joint may be reduced due to the lack of joint loading, and in addition, immobilization causes substantial changes in cartilage metabolism. The concomitant increase in MMP-9 and resistin concentrations during HDT bed rest may indicate the initiation of degenerative processes [41]. Concomitant changes in concentrations of different biomarkers may also indicate an interdependency at the molecular level. Both COMP and collagen II are substrates of several MMPs [7], and reduced serum COMP concentrations may be a direct result of reduced MMP activity, resulting in less COMP being released from the tissue.

YKL-40 did not show a significant response beyond the first measurement during recovery (R1), as indicated by an increase in YKL-40 concentrations. This may reflect local and systemic inflammatory activity during the initial days of reloading. It is possible that YKL-40 is not released from the cartilage matrix during degradation, but is produced by chondrocytes activated by an inflammatory response [42].

We observed decreasing serum concentrations for COMP, MMP-3, and YKL-40 already during the BDC period in some participants, which may be attributed to individual physical activity levels before the study (individual variations). This supports our previous findings of changes in type II collagen biomarkers during BDC [5], especially for serum C2C and serum C1,2C, both of which increased between BDC-3 and BDC-1. The participant-specific absolute changes in physical activity levels caused by moving to the clinic at the beginning of the study may substantially affect the magnitude of changes during the study and warrant further consideration in future bed rest studies to better understand the effect of pre-study lifestyle on musculoskeletal adaptation to bed rest.

Several studies have demonstrated direct effects of immobilization or hindlimb unloading (HLU) on cartilage health in rodents; both resulted in to cartilage degradation as evidenced by reductions in cartilage thickness [18], subchondral bone atrophy [18], altered chondrocyte density [43], and proteoglycan content [18,44,45] accompanied by increases in aggrecanases [18], expression of catabolic enzymes such as MMP-13 [44], oxidative stress [44], and COX-2 expression [43]. Furthermore, HLU was associated with an increase in MMP-3 mRNA expression, whereas moderate mechanical loading reduced MMP-3 staining and increased ADAMTS-5 expression [45]. These findings suggest that biomechanical forces may have a protective effect on cartilage. Additionally, research by Kwok et al. [44] demonstrated that exercise facilitates cartilage recovery. Another approach to prevent cartilage degeneration is the administration of nutritional supplements in rodent models. For example, administration of puerarin, a flavonoid isolated from the medicinal plant *Pueraria lobata*, has been shown to reduce the protein expression of cartilage-degrading enzymes and inflammatory factors, as well as to reverse type II collagen degradation in anterior cruciate ligament-transected rats, a standard model of OA [46]. These findings underscore the deleterious effects of immobilization or unloading on cartilage and highlight the potential protective effects of moderate exercise and nutritional supplementation. It should be noted that in rodent studies, the study duration can be shorter, and histochemical analysis is feasible whereas in healthy humans, such studies are only possible to a very limited extent. Bed rest studies longer than 21 days may provide a clearer picture of the effects of the interventions used. Additionally, optimizing the duration, intensity, frequency, or type of exercise intervention, as well as the dosage or type of nutritional supplementation, may yield stronger effects.

Comparing correlations from our unloading study with results from loading experiments, it is interesting to note that associations were detected between mechano-sensitive biomarkers such as COMP and MMP-3 in both scenarios [14,47]. In addition, we observed a positive correlation between MMP-9 and resistin, both biomarkers showing a significant increase during HDT bed rest. Our analysis revealed “moderate” associations between these biomarkers [48], similar to the findings of Mündermann et al. [47] in OA patients after a walking stress test, where both MMP-9 and resistin concentrations decreased in response to the walking stress. MMP-9 and resistin are known to be involved in the degradation of collagen and other ECM components [7,12]. These observations suggest that during immobilization (opposite to loading), biomarkers may be influenced by signaling pathways that upregulate inflammation and matrix turnover such as the CITED2-mediated pathway and NF-κB [49]. Previous studies have shown that bed rest leads to a decrease in tibial cartilage thickness [25] and an increase in biomarker concentrations of type II collagen degradation [5]. Therefore, we hypothesize that the combination of reductions in serum COMP and MMP-3 concentrations and increases in serum MMP-9 and resistin concentrations indicate a shift in the cartilage metabolism toward degradation in response to immobilization. However, when interpreting increased biomarker concentrations, it is important to consider that the headward fluid shift induced by the HDT position may contribute to these results. The fluid shift could disrupt focal adhesion formation, a process essential for chondrocytes to sense and respond to changes in their mechanical environment [50].

In our recent publication, we reported that type II collagen-related serum (CPII, C2C, and C1,2C) and urine (CTX-II, Coll2-1NO2) biomarkers indicate excess degradation and excretion of type II collagen [5] in the MNX study. This effect was not reversed upon re-ambulation and concentrations were still significantly different compared to baseline at R6 [5]. This is also true for MMP-9 in the present study, indicating an altered cartilage metabolism even after 6 days of recovery. The NeX intervention resulted in lower serum COMP concentrations at R6 compared to the CON, suggesting that vibration exercise plus whey protein/bicarbonate supplementation may influence the COMP concentration during the recovery phase. In this context, it is important to note that only 8 participants completed the NeX intervention, while 11 completed CON and all 12 completed RVE. Furthermore, no significant differences were observed for the NeX intervention at any other time point or for any other biomarkers. The RVE intervention alone had no effect on non-collagenous biomarker concentrations during HDT bed rest. This is consistent with our findings for type II collagen biomarkers, which were only affected by the NeX intervention [5]. The observed incomplete recovery of biomarkers to baseline concentrations suggests the need for longer post-study monitoring of these markers.

The major strength of the MNX study is the highly controlled study protocol and the examination of multiple serum biomarkers that reflect articular cartilage homeostasis. While the interventions were not explicitly designed to target cartilage degeneration, they were expected to prevent muscle degeneration and thus promote joint health. However, we have previously reported that RVE and NeX did not counteract thigh muscle atrophy in this study [32], probably due to the low training volume (twice a week for 25 ​min). This exercise protocol was predefined by ESA with the objective of identifying the minimum effective training load, but it appears that the chosen intensity was insufficient. These results could be specific to the Galileo vibration platform used in this study. This device provides an alternating vibration stimulus, unlike other commercially available platforms that typically provide a parallel vibration stimulus to both the right and left sides of the body.

In addition, no conclusions can be drawn about the nutrition intervention, as the authors did not have access to an isolated nutrition countermeasure. As only male participants were included in this study, no conclusions can be drawn about female cartilage metabolism, but this should be investigated in future studies. To assess the influence of the headward fluid shift on biomarker concentrations, a control group undergoing horizontal bed rest could be considered in future studies. Moreover, the concentrations of the selected serum biomarkers may not represent articular cartilage metabolism alone, as other tissues may contribute to these concentrations. However, the simultaneous measurement of multiple biomarkers may reduce this risk of misinterpretation.

In conclusion, our results demonstrate that immobilization exerts a significant influence on the serum concentrations of cartilage homeostasis biomarkers, indicating notable shifts in cartilage metabolism toward degenerative processes. Some of these changes persisted throughout the recovery period after bed rest, suggesting that the disruption of cartilage homeostasis induced by and maintained throughout immobilization may lead to progressive tissue degeneration that may be more pronounced the longer the immobilization lasts. The interventions did not show consistent effects, highlighting the need to identify exercise regimens with a clear protective effect on articular cartilage during periods of immobilization.

## Author contributions

Conception and design (AML, AN), analysis and interpretation of data (all authors), drafting of the article (AML, AN, MD), critical revision of the article for important intellectual content (all authors), final approval of the article (all authors), statistical expertise (ETG, KT, MD), obtaining of funding (AML, AN, GS, FZ), collection and assembly of data (AML, AN, MD, ND, MH).

## Declaration of generative AI and AI-assisted technologies in the writing process

All authors did not use generative AI and AI-assisted technologies in the writing process.

## Role of funding

This work was funded by the Federal Ministry for Economic Affairs and Climate Action, Germany (through German Aerospace Centre (DLR e.V.), German Space Agency (Project #: DLR 50WB0913, DLR 50WB1217, DLR 50WB1719, DLR 50WB1520, DLR 50WB2021, DLR 50WB2022)) and the European Space Agency and partially supported by the German Research Foundation (DFG) under Grant CRC 1483 EmpkinS-Project-ID 442419336 (AML, GS), Grant FOR 2722 – Project-ID 407168728, ZA 561/3-1 (FZ) and Grant FOR2722- Project-ID 407176282; NI 1083/4-1 (AN), BMBF-01EC1407A-MASCARA (AML, GS); ERC-2018-SyG: 4D+ nanoSCOPE – 810316 (AML, GS). The European Space Agency designed the study conditions of the MNX bed rest study (inclusion/exclusion criteria for participants, length of bed rest period, number of campaigns, design of the countermeasures). No study sponsor was involved in the collection, analysis, and interpretation of data; the writing of the manuscript; and in the decision to submit the manuscript for publication.

## Declaration of competing interest

None of the authors received any financial support or other benefits from commercial sources for the work reported in this manuscript.

## Figures and Tables

**figs1:**
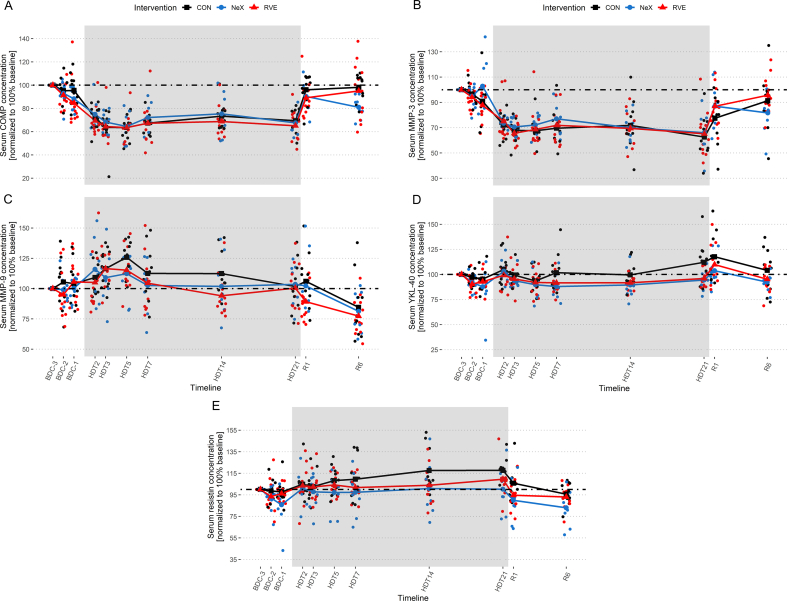


## References

[1] Kraus V.B., Blanco F.J., Englund M., Karsdal M.A., Lohmander L.S. (2015). Call for standardized definitions of osteoarthritis and risk stratification for clinical trials and clinical use. Osteoarthr. Cartil..

[2] Leong D.J., Sun H.B. (2014). Mechanical loading: potential preventive and therapeutic strategy for osteoarthritis. J. Am. Acad. Orthop. Surg..

[3] Lotz M., Martel-Pelletier J., Christiansen C., Brandi M.L., Bruyère O., Chapurlat R. (2013). Value of biomarkers in osteoarthritis: current status and perspectives. Ann. Rheum. Dis..

[4] Sandhu A., Rockel J.S., Lively S., Kapoor M. (2023). Emerging molecular biomarkers in osteoarthritis pathology. Therapeutic Advances in Musculoskeletal.

[5] Liphardt A.M., Godonou E.T., Dreiner M., Mündermann A., Tascilar K., Djalal N. (November 2023). Immobilization by 21 days of bed rest results in type II collagen degradation in healthy individuals. Osteoarthr. Cartil..

[6] Koelling S., Clauditz T., Kaste M., Miosge N. (2006). Cartilage oligomeric matrix protein is involved in human limb development and in the pathogenesis of osteoarthritis. Arthritis Res. Ther..

[7] Cabral-Pacheco G.A., Garza-Veloz I., Castruita-De La Rosa C., Ramirez-Acuna J.M., Perez-Romero B.A., Guerrero-Rodriguez J.F. (2020). The roles of matrix metalloproteinases and their inhibitors in human diseases. Indian J. Manag. Sci..

[8] Georgiev T., Ivanova M., Velikova T., Stoilov R. (2020). Serum levels of matrix metalloproteinase-3 as a prognostic marker for progression of cartilage injury in patients with knee osteoarthritis. Acta Reumatol. Port..

[9] Ramos-DeSimone N., Hahn-Dantona E., Sipley J., Nagase H., French D.L., Quigley J.P. (1999). Activation of matrix metalloproteinase-9 (MMP-9) via a converging plasmin/stromelysin-1 cascade enhances tumor cell invasion. J. Biol. Chem..

[10] Tizaoui K., Yang J.W., Lee K.H., Kim J.H., Kim M., Yoon S. (2022). The role of YKL-40 in the pathogenesis of autoimmune diseases: a comprehensive review. Int. J. Biol. Sci..

[11] Steppan C.M., Bailey S.T., Bhat S., Brown E.J., Banerjee R.R., Wright C.M. (2001). The hormone resistin links obesity to diabetes. Nature.

[12] Senolt L., Housa D., Vernerova Z., Jirasek T., Svobodova R., Veigl D. (2006). Resistin in rheumatoid arthritis synovial tissue, synovial fluid and serum. Ann. Rheum. Dis..

[13] Choe J.Y., Bae J., Jung H.Y., Park S.H., Lee H.J., Kim S.K. (2012). Serum resistin level is associated with radiographic changes in hand osteoarthritis: cross-sectional study. Jt. Bone Spine.

[14] Herger S., Vach W., Liphardt A.M., Nüesch C., Egloff C., Mündermann A. (2022). Experimental-analytical approach to assessing mechanosensitive cartilage blood marker kinetics in healthy adults: dose-response relationship and interrelationship of nine candidate markers. F1000Res.

[15] Liphardt A.M., Mündermann A., Andriacchi T.P., Achtzehn S., Heer M., Mester J. (2018). Sensitivity of serum concentration of cartilage biomarkers to 21-days of bed rest. J. Orthop. Res..

[16] Hinterwimmer S., Krammer M., Krötz M., Glaser C., Baumgart R., Reiser M. (2004). Cartilage atrophy in the knees of patients after seven weeks of partial load bearing. Arthritis Rheum..

[17] Vanwanseele B., Eckstein F., Knecht H., Spaepen A., Stüssi E. (2003). Longitudinal analysis of cartilage atrophy in the knees of patients with spinal cord injury. Arthritis Rheum..

[18] Nomura M., Sakitani N., Iwasawa H., Kohara Y., Takano S., Wakimoto Y. (2017). Thinning of articular cartilage after joint unloading or immobilization. An experimental investigation of the pathogenesis in mice. Osteoarthr. Cartil..

[19] Vanwanseele B., Lucchinetti E., Stüssi E. (2002). The effects of immobilization on the characteristics of articular cartilage: current concepts and future directions. Osteoarthr. Cartil..

[20] Primorac D., Molnar V., Rod E., Jelec Z., Cukelj F., Matisic V. (2020). Knee osteoarthritis: a review of pathogenesis and state-of-the-art non-operative therapeutic considerations. Genes.

[21] Guilak F. (2011). Biomechanical factors in osteoarthritis. Best Pract. Res. Clin. Rheumatol..

[22] Sun H.B. (2010). Mechanical loading, cartilage degradation, and arthritis. Ann. N. Y. Acad. Sci..

[23] Hovis K.K., Stehling C., Souza R.B., Haughom B.D., Baum T., Nevitt M. (2011). Physical activity is associated with magnetic resonance imaging–based knee cartilage T2 measurements in asymptomatic subjects with and those without osteoarthritis risk factors. Arthritis Rheum..

[24] Gahunia H.K., Pritzker K.P.H. (2012). Effect of exercise on articular cartilage. Orthop. Clin. N. Am..

[25] Liphardt A.M., Mündermann A., Koo S., Bäcker N., Andriacchi T.P., Zange J. (2009). Vibration training intervention to maintain cartilage thickness and serum concentrations of cartilage oligometric matrix protein (COMP) during immobilization. Osteoarthr. Cartil..

[26] de Sire A., Marotta N., Marinaro C., Curci C., Invernizzi M., Ammendolia A. (2021). Role of physical exercise and nutraceuticals in modulating molecular pathways of osteoarthritis. Int. J. Mol. Sci..

[27] Cermak N.M., Res P.T., De Groot L.C., Saris W.H., Van Loon L.J. (2012). Protein supplementation augments the adaptive response of skeletal muscle to resistance-type exercise training: a meta-analysis. Am. J. Clin. Nutr..

[28] Heer M., Baecker N., Frings-Meuthen P., Graf S., Zwart S.R., Biolo G. (2017). Effects of high-protein intake on bone turnover in long-term bed rest in women. Appl. Physiol. Nutr. Metabol..

[29] Jehle S., Hulter H.N., Krapf R. (2013). Effect of potassium citrate on bone density, microarchitecture, and fracture risk in healthy older adults without osteoporosis: a randomized controlled trial. J. Clin. Endocrinol. Metabol..

[30] Kakurin L.I., Lobachik V.I., Mikhailov V.M., Senkevich Y.A. (1976). Antiorthostatic hypokinesia as a method of weightlessness simulation. Aviat. Space Environ. Med..

[31] Hargens A.R., Vico L. (2016). Long-duration bed rest as an analog to microgravity. J. Appl. Physiol..

[32] Liphardt A.M., Bolte V., Eckstein F., Wirth W., Brüggemann G., Niehoff A. (2020). Response of thigh muscle cross-sectional area to 21-days of bed rest with exercise and nutrition countermeasures. Transl. Sports Med..

[33] Kenny H.C., Tascher G., Ziemianin A., Rudwill F., Zahariev A., Chery I. (2020). Effectiveness of resistive vibration exercise and whey protein supplementation plus alkaline salt on the skeletal muscle proteome following 21 Days of bed rest in healthy males. J. Proteome Res..

[34] Kermorgant M., Hammoud S., Mahieu L., Geeraerts T., Beck A., Bareille M.P. (2021). Effects of resistance exercise with or without whey protein supplementation on ocular changes after a 21-day head-down bed rest. Life.

[35] Guinet P., MacNamara J.P., Berry M., Larcher F., Bareille M.P., Custaud M.A. (2020). MNX (medium duration nutrition and resistance-vibration exercise) bed-rest: effect of resistance vibration exercise alone or combined with whey protein supplementation on cardiovascular system in 21-day head-down bed rest. Front. Physiol..

[36] Burnham K.P., Anderson D.R. (2004). Model Selection and Multimodel Inference.

[37] Nakagawa S., Johnson P.C.D., Schielzeth H. (2017). The coefficient of determination *R*^2^ and intra-class correlation coefficient from generalized linear mixed-effects models revisited and expanded. J. R. Soc. Interface.

[38] Koo T.K., Li M.Y. (2016). A guideline of selecting and reporting intraclass correlation coefficients for reliability research. J. Chiropractic Med..

[39] Hesselmann G. (2018). Applying linear mixed effects models (LMMs) in within-participant designs with subjective trial-based assessments of awareness—a caveat. Front. Psychol..

[40] Bakdash J.Z., Marusich L.R. (2017). Repeated measures correlation. Front. Psychol..

[41] Van Spil W.E., Szilagyi I.A. (2020). Osteoarthritis year in review 2019: biomarkers (biochemical markers). Osteoarthr. Cartil..

[42] Väänänen T., Koskinen A., Paukkeri E.L., Hämäläinen M., Moilanen T., Moilanen E. (2014). YKL-40 as a novel factor associated with inflammation and catabolic mechanisms in osteoarthritic joints. Mediat. Inflamm..

[43] Kaneguchi A., Ozawa J., Yamaoka K. (2022). Effects of joint immobilization and treadmill exercise on articular cartilage after ACL reconstruction in rats. Orthopaed. J. Sports Med..

[44] Kwok A.T., Mohamed N.S., Plate J.F., Yammani R.R., Rosas S., Bateman T.A. (2021). Spaceflight and hind limb unloading induces an arthritic phenotype in knee articular cartilage and menisci of rodents. Sci. Rep..

[45] Leong D.J., Gu X.I., Li Y., Lee J.Y., Laudier D.M., Majeska R.J. (2010). Matrix metalloproteinase-3 in articular cartilage is upregulated by joint immobilization and suppressed by passive joint motion. Matrix Biol..

[46] Ma T., Wen Y., Song X., Hu H., Li Y., Bai H. (2021). Puerarin inhibits the development of osteoarthritis through antiinflammatory and antimatrix-degrading pathways in osteoarthritis-induced rat model. Phytother Res..

[47] Mündermann A., Nüesch C., Herger S., Liphardt A.M., Chammartin F., De Pieri E. (2024). Load-induced blood marker kinetics in patients with medial knee compartment osteoarthritis are associated with accumulated load and patient reported outcome measures. F1000Res.

[48] Schober P., Boer C., Schwarte L.A. (2018). Correlation coefficients: appropriate use and interpretation. Anesth. Analg..

[49] Leong D.J., Hardin J.A., Cobelli N.J., Sun H.B. (2011). Mechanotransduction and cartilage integrity. Ann. N. Y. Acad. Sci..

[50] Hardy J.G. (2024). Articular cartilage loss is an unmitigated risk of human spaceflight. npj Microgravity.

