# Supplementary material for: Immobilization by 21-days of bed rest causes changes in biomarkers of cartilage homeostasis in healthy individuals
Source: Osteoarthr Cartil Open. 2025 Feb 28;7(2):100597. doi: 10.1016/j.ocarto.2025.100597 (PMC11938040; doi:10.1016/j.ocarto.2025.100597)
Supplement: Multimedia component 1 [file mmc1.docx]

Supplements

Table S1: Randomized filing of participants into interventions (CON, RVE and NeX) during the three campaigns, dropouts are crossed out (modified from Guinet et al., 2020 (reference 36)).

| Participants | Campaign 1  11/06/2012 to 12/16/2012 | Campaign 2  04/09/2013 to 05/19/2013 | Campaign 3  09/10/2013 to 10/20/2013 | |
| --- | --- | --- | --- | --- |
| 1 | **RVE** | **NeX** | | **CON** |
| 2 | **CON** | **RVE** | | **NeX** |
| 3 | **RVE** | **NeX** | | **CON** |
| 4 | **NeX** | **CON** | | **RVE** |
| 5 | **NeX** | **CON** | | **RVE** |
| 6 | **CON** | **RVE** | | **NeX** |
| 7 | **NeX** | **CON** | | **RVE** |
| 8 | **CON** | **RVE** | | **NeX** |
| 9 | **RVE** | **NeX** | | **CON** |
| 10 | **RVE** | **NeX** | | **CON** |
| 11 | **NeX** | **CON** | | **RVE** |
| 12 | **CON** | **RVE** | | **NeX** |

Table S2: Minimum detectable dose, intra- and inter-assay precision of the analyzed biomarkers.

| **Biomarker and manufacturer** | **Minimum detectable dose** | **Intra-assay precision/ coefficient of variation (CV%)** | **Inter-assay precision (CV%)** |
| --- | --- | --- | --- |
| AnaMar COMP® ELISA | <0.1 U/L | 4.0 | 8.5-13.6 |
| Human Total MMP-3 Quantikine^TM^ ELISA Kit | 0.002‐0.045 ng/mL | 2.9 | 7.0-8.6 (manufacturer) |
| Human MMP-9 Quantikine^TM^ ELISA Kit | <0.156 ng/mL | 2.0 | 6.9-7.9 (manufacturer) |
| MicroVue^TM^ Bone YKL40 EIA | 5.4 ng/mL | 4.6 | 6.2-6-6 |
| Human Resistin Quantikine® ELISA | 0.010-0.055 ng/mL | 2.0 | 7.8-9.2 (manufacturer) |

Table S3: Results of repeated measures correlation (rmCorr) analysis between biomarkers. Including lower and upper 95% confident interval (CI), unadjusted p-values and p-values adjusted for multiple comparison using False Discovery Rate (FDR).

| **Biomarkers** | | **rmCorr** | **lower CI** | **upper CI** | **unadjusted p-values** | **FDR (adjusted p-values)** |
| --- | --- | --- | --- | --- | --- | --- |
| COMP | MMP-3 | 0.579 | 0.503 | 0.647 | <0.001 | **<0.001** |
| COMP | MMP-9 | -0.255 | -0.354 | -0.151 | <0.001 | **<0.001** |
| COMP | resistin | -0.110 | -0.216 | -0.002 | 0.046 | 0.057 |
| COMP | YKL-40 | 0.117 | 0.009 | 0.222 | 0.035 | **0.049** |
| MMP-3 | MMP-9 | -0.095 | -0.200 | 0.012 | 0.082 | 0.091 |
| MMP-3 | resistin | -0.150 | -0.252 | -0.044 | 0.006 | **0.010** |
| MMP-3 | YKL-40 | -0.012 | -0.119 | 0.095 | 0.821 | 0.821 |
| MMP-9 | resistin | 0.580 | 0.505 | 0.647 | <0.001 | **<0.001** |
| MMP-9 | YKL-40 | 0.199 | 0.094 | 0.300 | <0.001 | **<0.001** |
| resistin | YKL-40 | 0.347 | 0.249 | 0.438 | <0.001 | **<0.001** |

Table S4: Results of Linear Mixed Models (LMM) to examine the relationship between the changes in COMP concentration and matrix metalloproteinase (MMP)-3, MMP-9, YKL-40 or resistin over time (predictors). Adjusted for “interventions” as covariates. CI: 95% confidence interval, CON: control intervention, Conditional R^2^: considers both fixed and random effects, ICC: intraclass correlation coefficient, marginal R^2^: variance of the fixed effects, N: number of participants, NeX: nutrition and RVE intervention, RVE: resistive vibration exercise intervention, Ref.: Reference level.

|  | **COMP** | |
| --- | --- | --- |
| *Predictors* | *Estimates [CI]* | *p* |
| (Intercept) | 2.52 [1.00, 4.04] | **0.001** |
| MMP-3 | 0.27 [0.23, 0.30] | **<0.001** |
| MMP-9 | -0.004 [-0.005, -0.002] | **<0.001** |
| YKL-40 | 0.03 [0.01, 0.04] | **<0.001** |
| Resistin | 0.02 [-0.08, 0.12] | 0.634 |
|  |  |  |
| Intervention |  |  |
| Intervention [CON] | Ref. |  |
| Intervention [NeX] | 0.29 [0.06, 0.52] | **0.012** |
| Intervention [RVE] | 0.38 [0.18, 0.58] | **<0.001** |
|  |  |  |
| **Random effects** | | |
| ICC | 0.89 | |
| N _Subject_ | 12 | |
| Observations | 337 | |
| Marginal R^2^ / Conditional R^2^ | 0.331 / 0.927 | |

Figure S1: Serum concentrations normalized to baseline (BDC-3, 100%) over time for COMP (A), MMP-3 (B), MMP-9 (C), YKL-40 (D) and resistin (E). Mean by intervention over time (colored solid lines: intervention means; colored dots: individual values of subjects). The grey area indicates the bed rest period. BDC: baseline data collection; CON: control intervention; HDT: head-down-tilt bed rest; RVE: resistive vibration exercise; NeX: nutrition and RVE; R: recovery.
